# High EVI1 and PARP1 expression as favourable prognostic markers in high-grade serous ovarian carcinoma

**DOI:** 10.1186/s13048-023-01239-6

**Published:** 2023-07-31

**Authors:** Paul Jank, Jonas Leichsenring, Svenja Kolb, Inga Hoffmann, Philip Bischoff, Catarina Alisa Kunze, Mihnea P. Dragomir, Moritz Gleitsmann, Moritz Jesinghaus, Wolfgang D. Schmitt, Hagen Kulbe, Christine Sers, Albrecht Stenzinger, Jalid Sehouli, Ioana Elena Braicu, Christina Westhoff, David Horst, Carsten Denkert, Stefan Gröschel, Eliane T. Taube

**Affiliations:** 1 Institute of Pathology, Philipps-University Marburg, University Hospital Marburg (UKGM), Marburg, Germany; 2 Institute of Pathology, Zytologie Und Molekulare Diagnostik, REGIOMED, Klinikum Coburg, Coburg, Germany; 3 Department of Gynecology, Vivantes Netzwerk Für Gesundheit GmbH Berlin, Vivantes Hospital Neukölln, Rudower Straße 48, 12351 Berlin, Germany; 4 Institute of Pathology, Charité Universitätsmedizin Berlin, corporate member of Freie Universität Berlin and Humboldt Universität Zu Berlin, CCM, Charitéplatz 1, 10117 Berlin, Germany; 5 Tumorbank Ovarian Cancer Network, Charité, Universitätsmedizin Berlin, corporate member of Freie Universität Berlin, Humboldt-Universität Zu Berlin, and Berlin Institute of Health, 10117 Berlin, Germany; 6 Department of Gynecology, European Competence Center for Ovarian Cancer, Charité Universitätsmedizin Berlin, corporate member of Freie Universität Berlin, Humboldt-Universität Zu Berlin, and Berlin Institute of Health, 10117 Berlin, Germany; 7 Institute of Pathology, University Hospital Heidelberg, Heidelberg, Germany; 8 Oncology Center Worms, Worms, Germany

**Keywords:** Ovarian cancer, Biomarker, Prognostic biomarker, HGSOC, EVI1, PARP

## Abstract

**Background:**

Mechanisms of development and progression of high-grade serous ovarian cancer (HGSOC) are poorly understood. EVI1 and PARP1, part of TGF-ß pathway, are upregulated in cancers with DNA repair deficiencies with DNA repair deficiencies and may influce disease progression and survival. Therefore we questioned the prognostic significance of protein expression of EVI1 alone and in combination with PARP1 and analyzed them in a cohort of patients with HGSOC.

**Methods:**

For 562 HGSOC patients, we evaluated EVI1 and PARP1 expression by immunohistochemical staining on tissue microarrays with QuPath digital semi-automatic positive cell detection.

**Results:**

High EVI1 expressing (> 30% positive tumor cells) HGSOC were associated with improved progression-free survival (PFS) (HR = 0.66, 95% CI: 0.504–0.852, *p* = 0.002) and overall survival (OS) (HR = 0.45, 95% CI: 0.352–0.563, *p* < 0.001), including multivariate analysis. Most interestingly, mutual high expression of both proteins identifies a group with particularly good prognosis. Our findings were proven technically and clinically using bioinformatical data sets for single-cell sequencing, copy number variation and gene as well as protein expression.

**Conclusions:**

EVI1 and PARP1 are robust prognostic biomarkers for favorable prognosis in HGSOC and imply further research with respect to their reciprocity.

**Supplementary Information:**

The online version contains supplementary material available at 10.1186/s13048-023-01239-6.

## Introduction

Although ovarian carcinoma (OC) has a relatively low incidence (worldwide 3.4%), it is among the five top leading causes of female cancer deaths in the US [1, 2]. High-grade serous ovarian carcinoma (HGSOC) is the most frequent and most aggressive histological subtype [3].

HGSOC is characterized by early *TP53* mutations resulting in high chromosomal instability. A predisposing condition is homologous recombination deficiency (HRD) including *BRCA1/2* mutations or BRCAness conditions. Reported rates of germline or somatic *BRCA* 1/2 mutations in epithelial ovarian carcinoma vary from approximately 20% in literature, if only HGSOC are considered to 59% [4–6]. *BRCA 1/2* constitute two genes of a large gene-network providing a repair mechanism for double strand breaks in the DNA, known as homologous recombinational repair (HRR). Apart from *BRCA 1/2*, other genes may also perturb HRR, resulting in HRD. Full scope testing of these genes is difficult, therefore indirect evidence of HRD, such as loss of heterozygosity (LOH), telomeric allelic imbalance (TAI) and large-scale transitions, (LST) is integrated into a HRD signature summarized as genomic scar testing [5, 7]. Prevalence of HRD occurs in up to 70% of OC [8]. The predictive value of HRD testing is controversial. However, single strand DNA repair mechanisms such as PARP are often upregulated in HGSOC and the suppression of alternative DNA repair mechanisms via Poly (ADP-ribose) polymerase (PARP1/2*)* inhibitors (PARPi) shows a significant improvement for patients with HGSOC overall but especially when HR deficient and have led to FDA approval in first-line and recurrent setting [9, 10]. In the homologous repair pathway different genes (e.g., *BRCA*) and Fanconi anemia proteins are interacting in DNA repair, especially in OC [11]. The oncogene *EVI1* was first discovered in myeloid malignancies [12] and alterations of the corresponding chromosomal region 3q26-29 are associated with disease progression in Fanconi anemia [12, 13]. *EVI1* overexpression in ovarian carcinoma cell lines has previously been described [14]. Functionally, EVI1 is a dual-domain zinc finger transcription factor that regulates different cancer genes or genes associated with cancer [15]. It is involved in proliferation, regulation of cell cycle progression as well as inhibition of apoptosis and differentiated cellular growth response [16, 17]. The exact pathways and mechanisms are not understood in detail yet, but inhibition of the *TGF-b* pathway via SMAD3 may play a role in apoptosis [17–19]. Furthermore, the alteration of EVI1 in fallopian tube epithelium has been previously described and suggests an early event in cancer genesis [20, 21]. A physical crosstalk between *PAX8,* commonly expressed in tubal epithelium, and the gene locus of EVI1 (*MECOM*) was investigated, using several ovarian cancer cell lines [20].

EVI1 is best known for its prominent roles in myeloid neoplasms, especially in acute myeloid leukemia (AML) with chromosome 3 rearrangements [22, 23]. EVI1 overexpression is an independent factor for worse survival in all subtypes of AML [24, 25]. Furthermore, PARP1 is upregulated and a therapeutic target in EVI1-deregulated AML with chromosomal 3q21q26 aberrations (3q-AML) [26]. Since EVI1 is upregulated in certain cancer types with chromosomal instabilities and since single strand DNA repair mechanisms such as PARP1 are often upregulated in HGSOC, we investigated the frequency and prognostic importance of EVI1 and PARP1 in HGSOC [26, 27]. We measured protein level by quantitative analysis of immunohistochemical expression in our in-house cohort of 562 patients and tested its association with survival. Furthermore, we used several bioinformatical data sets of the Kaplan–Meier plotter (KMp), cBioPortal (cBio) and single-cell sequencing data to evaluate *EVI1* and *PARP1* expression on chromosomal, mRNA and protein level and their influence on patients’ prognosis.

## Material and methods

### Study population

Formalin-fixed and paraffin-embedded (FFPE) primary tumor tissue of 562 HGSOC patients diagnosed between 1991 and 2013 were retrospectively analyzed to determine the influence of EVI1 and PARP1 protein expression on pathological disease parameters and survival. Patients were treated at the Clinic of Gynecology Charité Campus Virchow Clinic in Berlin and diagnosed according to WHO criteria [3] at the Institute of Pathology, Charité Berlin, reviewed by experienced gyneco-pathologists. Since the Clinic of Gynecology is a certified European gynecologic competence center for ovarian cancer, debulking surgery is performed by specialists for complete resection, followed by systemic therapy according to appropriate guidelines.

The patients have been followed from 1994 to 2021 and their clinical data were obtained from the Tumor Bank Ovarian Cancer Network (TOC: www.toc-network.de) or the Charité Comprehensive Cancer Center (CCCC: https://cccc.charite.de). The study was performed in accordance with the Declaration of Helsinki and with local ethical guidelines (ethic committee approval number EA1/051/18) and is supported by the TRANSCAN-2 project (grant no.: 2014–121). Written patient’s consent was given within treatment agreement of the Charité University-Hospital Berlin. Treatment agreement forms from 2005 and 2021 can be obtained in the supplementary material.

Patients’ baseline parameters, according to EVI1 expression status, are summarized in Table 1.

**Table 1:** Baseline characteristics of study cohort, stratified by EVI1 protein expression

**Category**		**All **	**EVI1 low (≤ 30%)**	**EVI1 high(> 30%)**	***p*-value**
**All**		562 (100.0)	130 (23.1)	432 (76.9)	--
**Age**	**≤ 60**	263 (48.0)	54 (43.2)	209 (49.4)	0.262
	**>60**	285 (52.0)	71 (56.8)	214 (50.6)	
	missing	14 (2.5)			
**Tumor stage**	**pT1**	37 (6.7)	4 (3.2)	33 (7.7)	0.123
	**pT2**	40 (7.2)	7 (5.6)	33 (7.7)	
	**pT3**	475 (86.1)	114 (91.2)	361 (84.6)	
	missing	10 (1.8%)			
**Nodal stage**	**pN0**	145 (32.3)	37 (36.3)	108 (31.1)	0.400
	**pN+**	304 (67.7)	65 (63.7)	239 (68.9)	
	missing	113 (20.1%)			
**FIGO group**	**FIGO I**	29 (5.2)	4 (3.2)	25 (5.8)	0.072
	**FIGO II**	28 (5.0)	5 (4.0)	23 (5.4)	
	**FIGO III**	392 (70.8)	88 (69.8)	304 (71.0)	
	**FIGO IV**	105 (19.0)	29 (23.0)	76 (17.8)	
	missing	8 (1.4)			
**PARP1**	**Low **(≤ 45%)	266 (56.8)	70 (66.0)	196 (54.1)	0.019
	**High **(> 45%)	202 (43.2)	36 (34.0)	166 (45.9)	
	missing	94 (16.7)			
**Chemotherapy**	**Platinum + Taxol**	413 (94.3)	89 (94.7)	324 (94.2)	0.984
	**Platinum + other**	6 (1.4)	1 (1.1)	5 (1.5)	
	**Platinum alone**	13 (3.0)	3 (3.2)	10 (2.9)	
	**Other**	1 (0.2)	0 (0)	1 (0.3)	
	**No chemotherapy**	5 (1.1)	1 (1.1)	4 (1.2)	
	missing	124 (22.1)			

N (percent of valid cases without missings) within categorical variable

### Immunohistochemical staining and computational pathology analysis

Using a tissue microarray (TMA, 1.5 mm diameter, one core per patient) with representative tumor tissue from each patient, EVI1 staining was performed at the Institute of Pathology in Heidelberg, with 1:2500 diluted Cell Signaling anti-EVI1 (#2593, clone C50E12) antibody, after EDTA antigen retrieval (pH = 9), on a Ventana staining system. PARP1 staining was performed at the Institute of Pathology, Charité Berlin, with the Ventana staining system, using anti-PARP1 (#sc-8007, clone F-2, Santa Cruz Biotechnology inc.) antibody in a 1:100 dilution and CC1 Tris–EDTA (pH = 7.8) antigen retrieval. EVI1 stained slides were digitized with Hamamatsu Slide Scanner, while PARP1 stained slides were scanned using Panoramic Slide Scanner (3DHISTECH). Using the open-source software QuPath (Version 0.1.2), we determined the positive nuclear cell staining of EVI1 and PARP1 with parameters described in Tab. S1.

**Fig. 1:**
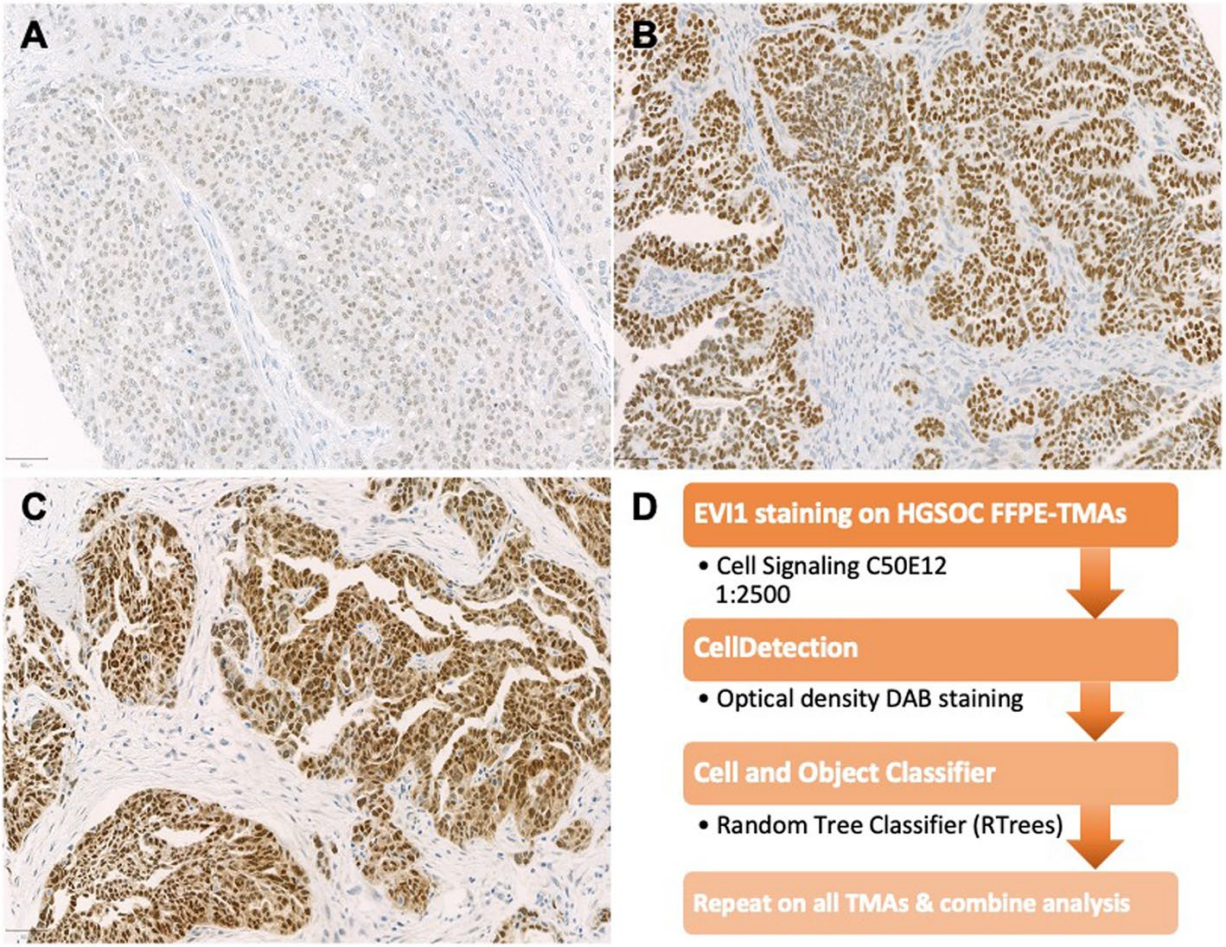
EVI1 staining on HGSOC FFPE tumor tissue and image analysis workflow using semi-automatically QuPath software. (**A**-**C**) different EVI1 expression profiles in 20 × magnification (**D**) EVI1 assessment workflow

By teaching the object classifier (“RTrees” random tree method, maximum tree number = 50, minimum sample count = 10), done by J.L. and S.K. under the guidance of board approved gynecological pathologist (E.T.T.), the staining was determined between invasive tumor cells versus tumor stroma cells. QuPath analysis delivered proportions of positively stained tumor cells for each TMA core. Cutoff values for binary classification (EVI1 low and high, PARP1 low and high) were determined by using “Cutoff Finder” (CF, https://molpathoheidelberg.shinyapps.io/CutoffFinder_v1/) [28]. A summary of EVI1 protein assessment is shown in Fig. 1

### In-silico analysis using public bulk gene expression data

We used the Kaplan–Meier plotter (https://kmplot.com/analysis/index.php?p=background) to examine the prognostic impact of *EVI1* and *PARP1* mRNA expression regarding patients’ outcome [28, 29]. The Kaplan–Meier Plotter uses datasets established before 2012, while high- and low-grade serous carcinomas were recognized as different entities by the WHO classification in 2014. Thus, the categories low- and high-grade serous carcinomas are not eligible. As an approximation, we selected serous carcinomas grade 2 + 3 since they most likely fulfill the desired diagnostic criteria of growth pattern, pleomorphy and mitotic rate. Affymetrix mRNA data were available for *n* = 1029 for PFS and *n* = 1144 for OS. Within cBioPortal (https://www.cbioportal.org), TCGA Ovarian Serous Cystadenocarcinoma was chosen, source data from GDAC Firehose, previously known as TCGA Provisional. We examined correlations between *EVI1* and *PARP1* DNA copy number variation (CNV), mRNA expression as well as correlation on protein level, using a cohort of *n* = 617 HGSOC patients. For common pathway analysis, we used the DAVID tool as well as functional annotation charts [30].

### Single-cell gene expression analysis in two publicly available HGSOC datasets

Single-cell gene expression data and metadata of two public datasets [31, 32] were downloaded and processed using the open-source software “R” (Version 4.1.1) and the package “Seurat” (version 4.1.0) [33]. Low-quality transcriptomes were excluded by filtering for cells containing 500–6,000 genes, 1000–60,000 reads, and < 20% mitochondrial reads. Gene expression was normalized using the scTransform function. Cells were clustered utilizing the first 10 principal components at a resolution of 0.2 and visualized by uniform manifold approximation and projection (UMAP). Cell types were annotated according to mean expression of canonical cell type marker genes per cluster. Gene expression of *EVI1* was visualized using the FeaturePlot and VlnPlot functions.

### Statistical methods

The statistical analysis was conducted using SPSS Statistics Version 27.0.0.0 64-Bit (IBM, Armonk, USA). For categorical variables, we determined crosstab *p*-values with Fisher’s exact test in a 2 × 2 matrix. For matrices > 2 × 2, the Chi^2^ test was used.

We used the “Cutoff Finder” web application (CF) to stratify patients into groups of high and low biomarker expression, according to EVI1 and PARP1 expression [28]. For both immunohistochemical markers, percentage values of positively stained tumor cells as well as PFS and OS were used to determine optimal cutoffs (best *p*-value using log-rank tests) for binary biomarker classification with highest differences in median survival. According to the Bonferroni method, the range of optimal cut-offs was set to at least 5% to reduce type 2 errors. CF delivered 30.38% positively stained tumor cells as optimal cut-off (best *p*-value) for EVI1, while almost all cut-offs seemed significant (460 out of 491, 93.1%). For practicality, the cut-off was set to 30%. For PARP1, CF determined 46.39% positively stained tumor cells as best cut-off for OS, with at least 25% (116 out of 463) of cut-offs significant. Following the same reasoning as with EVI1, we used 45% as cut point for PARP1 in further analysis.

Survival analysis was done using Kaplan–Meier plots with log-rank tests for classifying differences in median survival as well as uni- and multivariate Cox proportional hazards models with binary biomarker category predicting survival endpoints. Progression-free survival (PFS) was defined as time to event, determined by clinical and imaging examination. Events for overall survival (OS) were defined as death irrespective of cause. Median follow-up time was for PFS = 17.51 months and for OS = 34.52 months. Statistically significant cases were at a *p*-value ≤ 0.05 while using a 95% confidence interval (CI).

## Results

### Baseline characteristics of study cohort

We investigated a cohort of 562 patients with HGSOC. Patients were older than 60 years at diagnosis in 285 (52.0%) cases. Most HGSOC patients were staged as pT3 with 475 (86.1%) cases, followed by 40 (7.2%) patients with a pT2 stage tumor and 37 (6.7%) patients with a pT1 stage cancer burden. In our cohort, an enrichment for high FIGO stages could be detected: International Federation of Gynecology and Obstetrics (FIGO) III ovarian cancer burden was seen in 392 patients (70.8%) followed by FIGO IV (105 patients, 19.0%) and FIGO I (29 patients, 5.3%) and II (28 patients, 5.1%). 304 (67.8%) patients had node-positive disease at time of diagnosis. Most patients received a combined Platinum- and Taxol-based chemotherapy. Exact values can be found in Table 1..

### Immunohistochemical protein expression of EVI1 and PARP1

**Fig. 2:**
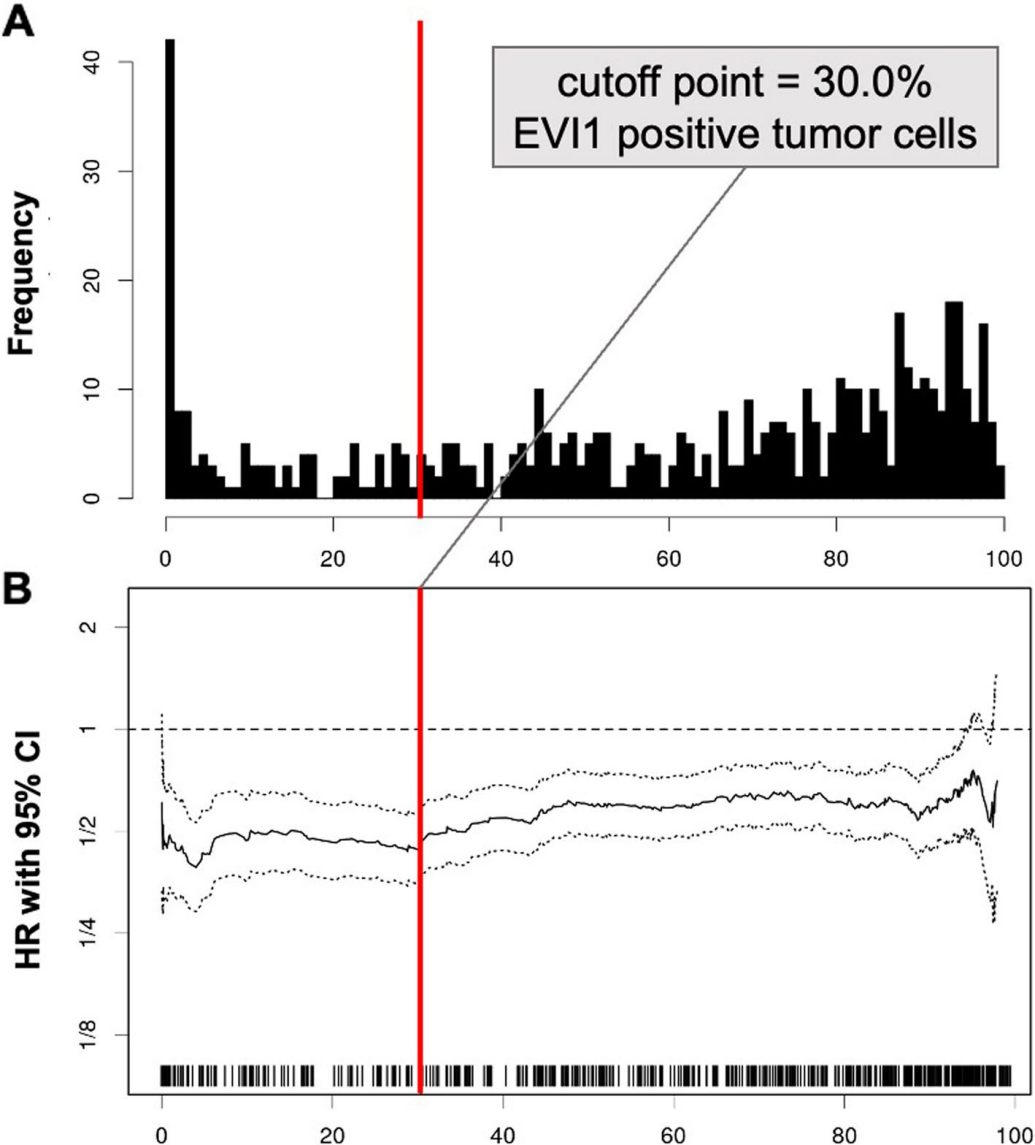
Systematic analysis for optimal cut-off for EVI1 expression on tumor cells as a prognostic marker in HGSOC. (**A**) Frequency of EVI values, (**B**) Hazard ratio (HR) and 95% confidence intervals (CI) of continuous EVI1 expression using overall survival in biostatistical tool *Cutoff Finder*

EVI1 was almost exclusively expressed in the nucleus with strong intensity in tumor cells. Overall staining pattern was clearly evaluable for a trained pathologist, as seen in Fig. 1A-C. We used computer assisted evaluation, determining positive cell counts with high precision (Fig. 1D). Figure 2A shows a histogram with detected percent values and Fig. 2B the corresponding hazard ratio (HR) for each cut-off point.

For EVI1, a significant cut-off could be defined in 460 out of 491 analyzed cut-offs. As a potentially applicable cut-off for daily routine, we chose 30% of stained tumor cells resulting in EVI1 low ≤ 30% (130 patients, 23.1%) and EVI1 high > 30% (432 patients, 76.9%).

PARP1 expression was also nuclear, but with different intensities and in some cases with artificial cytoplasmic staining. The Cutoff determined for PARP1 was 45%, dividing our cohort in PARP1 low ≤ 45% (266 patients, 56.8%) and PARP1 high > 45% (202 patients, 43.2%) (Suppl. Figure 1). Since TMA material for 94 (16.7%) patients were consumed, no PARP1 data could be obtained.

### EVI1 protein expression and its prognostic impact

The two EVI1 groups (low, high) were well balanced regarding classical pathological risk parameters, such as tumor staging, nodal stage, grading or FIGO status. No significant differences were noticed (Table 1.). However, we found a correlation between positive EVI1 protein expression and higher rates of patients with high PARP1 protein expression (*p* = 0.019).

Determining median OS in the full cohort of *n* = 544, we found considerable differences between the two groups: EVI1 low patients (*n* = 125) with a median OS of 23.40 months (95% CI 20.57–26.21) versus EVI1 high cohort (*n* = 419) with 48.59 months (95% CI 43.39–53.79), resulting in double the survival time for the EVI1 high cohort (*p* < 0.001). Interestingly, we found patients with very long survival in the EVI1 high protein expression group.

Similar results were seen for PFS Kaplan–Meier curves (with log-rank test), indicating a difference in median PFS in the overall group: EVI1 low group (*n* = 87) with 16.46 months (95% CI 14.47–18.45) and EVI1 high group (*n* = 330) with 20.70 months (95% CI 18.01–23.29) median survival, with *p* = 0.001.

Using Cox proportional hazards models for both survival endpoints, a high EVI1 protein expression led to significantly improved OS (HR = 0.445, 95% CI: 0.352–0.563, *p* < 0.001) and PFS (HR = 0.655, 95% CI: 0.504–0.852, *p* = 0.002) prediction. Using typical prognostic factors (complete resection according to surgeon [R status], age at diagnosis, FIGO) within multivariate Cox-regression models, EVI1 high proved to be an independent prognostic factor for survival (for OS: *n* = 425, HR = 0.480, 95% CI 0.369–0.624, *p* < 0.001; for PFS: *n* = 354, HR = 0.700, 95% CI 0.527–0.929, *p* = 0.013).

**Fig. 3:**
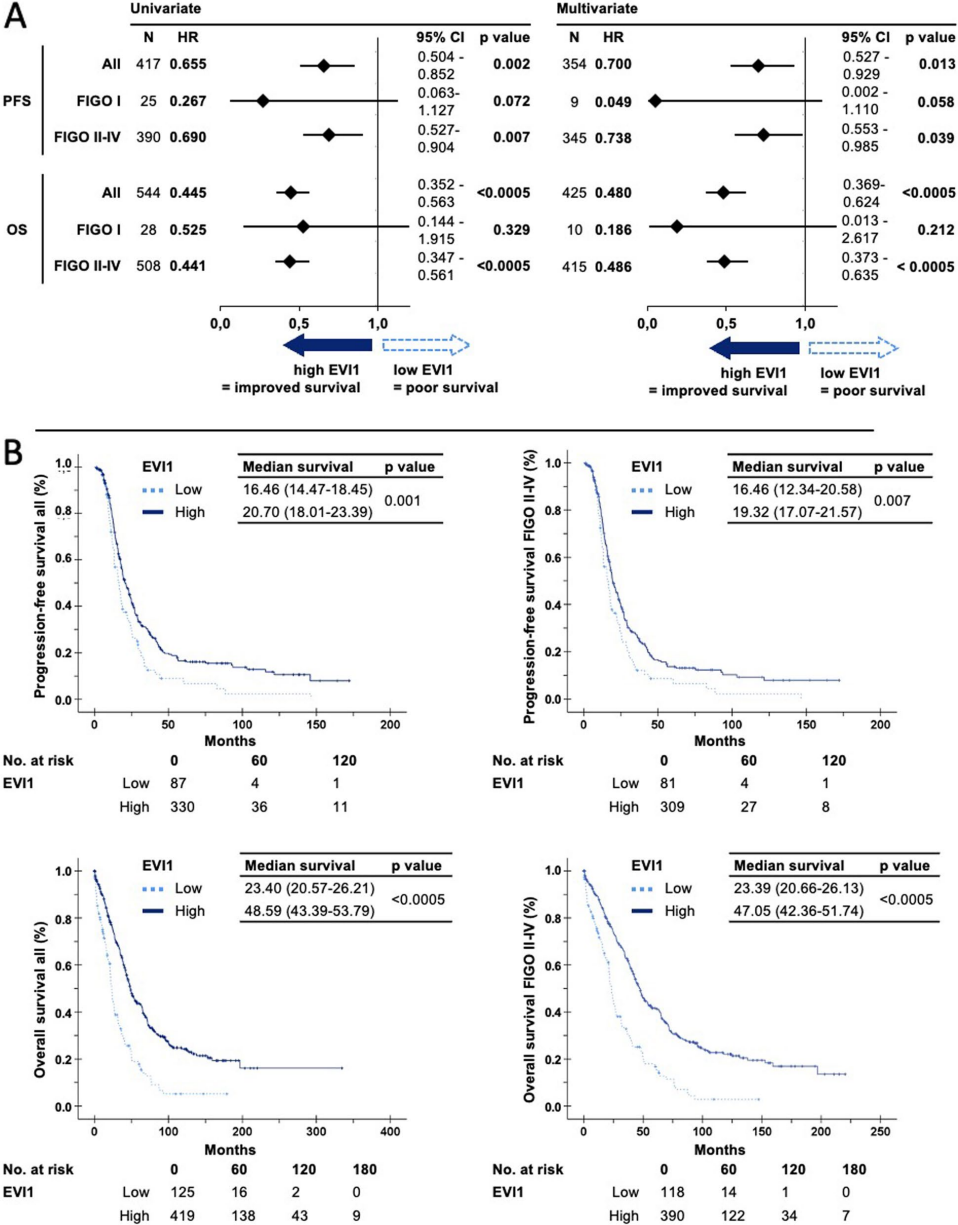
Survival analysis in EVI1 low and high HGSOC tumors, with and without FIGO stratification. (**A**) Uni- and multivariate cox proportional hazards models with binary biomarker category (**B**) Kaplan–Meier plots and differences in median survival

To test the prognostic impact of EVI1 with respect to FIGO status, we assessed Kaplan–Meier curves as well as cox proportional hazard models for each FIGO stage. As FIGO II-IV cases have a similar clinical behavior regarding survival and EVI1 expression, we divided the cohort in FIGO II-IV versus FIGO I patients. In FIGO II–IV HGSOC patients, a similar trend regarding survival was seen: FIGO II-IV group univariate analysis yielded an OS HR = 0.441 (95% CI 0.347–0.561, *p* < 0.001) and PFS HR = 0.690 (95% CI 0.527–0.904, *p* = 0.007), and FIGO II-IV multivariate analysis: OS HR = 0.486 (95% CI 0.373–0.635, *p* < 0.001) and PFS HR = 0.738 (95% CI 0.553–0.985, *p* = 0.039). For cases with FIGO I status, EVI1 tended to have no prognostic impact. Figure 3A shows the results of the regression models illustrated as forest plots, while Fig. 3B presents Kaplan–Meier curves with median survival.

### PARP1 protein expression within the EVI1 context

High PARP1 protein expression was predicting lower rates of survival events in the overall cohort and especially in the FIGO II-IV stage subgroup, using uni- and multivariate Cox proportional hazards models. Multivariate analysis in the overall cohort: PFS with HR = 0.610 (95% CI 0.465–0.799, *p* < 0.001), multivariate OS with HR = 0.667 (95% CI 0.511–0.871, *p* = 0.003). FIGO II-IV stage PFS with HR = 0.631 (95% CI 0.480–0.830, *p* = 0.001) and FIGO II-IV OS with HR = 0.688 (95% CI 0.527–0.899, *p* = 0.006). Detailed results are shown in Suppl. Figure 2.

**Fig. 4:**
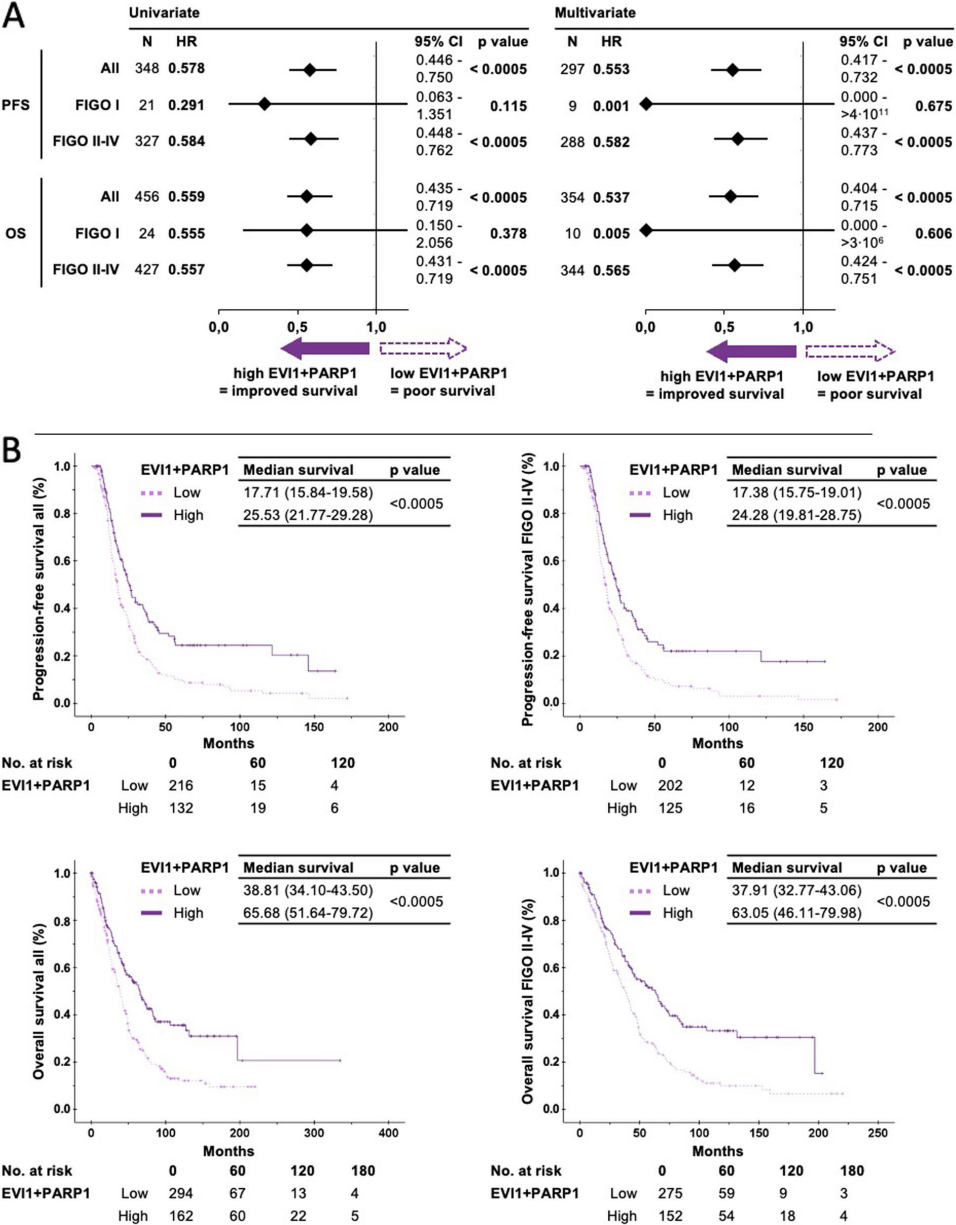
Survival analysis in EVI1-PARP1 low (EVI1-PARP1 + , EVI1 + PARP1- and EVI1-PARP1-) and high (EVI1 + PARP1 +) HGSOC tumors, with and without FIGO stratification. (**A**) Uni- and multivariate cox proportional hazard models with binary biomarker category (**B**) Kaplan–Meier plots and differences in median survival

By using the binary classification for each biomarker, we combined both values to PARP1-EVI1 high/high, high/low, low/high and low/low and generated Kaplan–Meier plots for PFS and OS. As evident in Suppl. Figure 3, improved PFS and OS were seen in PARP1-EVI1 high/high (EVI1^+^PARP1^+^) compared to all other groups (EVI1^−^PARP1^+^, EVI1^+^PARP1^−^ and EVI1^−^PARP1^−^). Therefore, we defined this constellation as a new binary variable and applied Cox proportional hazard models. Improved survival endpoints were predicted for the EVI1^+^PARP1^+^ group versus the three other combinations: multivariate overall OS HR = 0.537 (95% CI 0.404–0.715, *p* < 0.001), overall PFS HR = 0.553 (95% CI 0.417–0.732, *p* < 0.001), FIGO II-IV OS HR = 0.565 (95% CI 0.424–0.751, *p* < 0.001) and FIGO II-IV PFS HR = 0.582 (95% CI 0.437–0.773, *p* < 0.001) (Fig. 4).

### In silico analysis for EVI1 and PARP1 using bioinformatical datasets

Using Kaplan–Meier plotter (KMp), we examined the prognostic impact of *EVI1* mRNA expression on PFS (*n* = 1029 patients) and OS (*n* = 1144 patients) in bioinformatical datasets. Since the KMp did not identify probes for *EVI1* with best fit according to JetSet [34] standard, we used three independent probes (208434_at, 215851_at, 221884_at) with moderately good fitting, as shown in Suppl. Figure 4. Data analysis showed differences in survival with respect to *EVI1* mRNA expression (PFS best result with (probeID 221884_at): HR = 0.71 [95% CI 0.61–0.82], *p* < 0.001) and OS best result with (probeID 208644_at): HR = 0.79 [95% CI 0.67–0.94] *p* = 0.0064).

Using probe 298644_at (best JetSet fit) for *PARP1* mRNA expression analysis, we found no prognostic impact on PFS (HR = 1.18 [95% CI 0.99–1.4], *p* = 0.063) and OS (HR = 1.11 [95% CI 0.92–1.33], *p* = 0.270).

Furthermore, we evaluated connections between *EVI1* and *PARP1* on DNA and mRNA levels regarding mutual pathway overlaps. By using cBioPortal, we did not detect any coherence on copy number variation (CNV): Spearman: 0.06, *p* = 0.156 (Suppl. Figure 5A). On mRNA level, *EVI1* and *PARP1* had a Spearman correlation factor of 0.31, with a *p*-value of 2.98 × 10^–8^ (Suppl. Figure 5B). Moreover, we detected a correlation on protein level, using open-source data with different detection methods. PARP1 protein level (mass spectrometry [MS] by The Clinical Proteomic Tumor Analysis Consortium [CPTAC]) and EVI protein level (MS) revealed a moderately high correlation: Spearman = 0.51 (*p* = 2.94 × 10^–12^) (Suppl. Figure 5C-D). Pathway analyses done in DAVID Bioinformatics Resources found no common pathway containing both of the genes [30]. However, Functional Annotation Chart for PARP1 und EVI1, shown in Suppl. Figure 5E, indicates that the two genes contribute to the MOTIF processes of nuclear localization signal (*p* = 0.020) and to apoptosis (*p* = 0.032), although the adjusted *p*-values for multiple testing correction by using the Benjamini and Hochberg method were not significant (*p* = 1.0).

### Single-cell analysis of public datasets

**Fig. 5:**
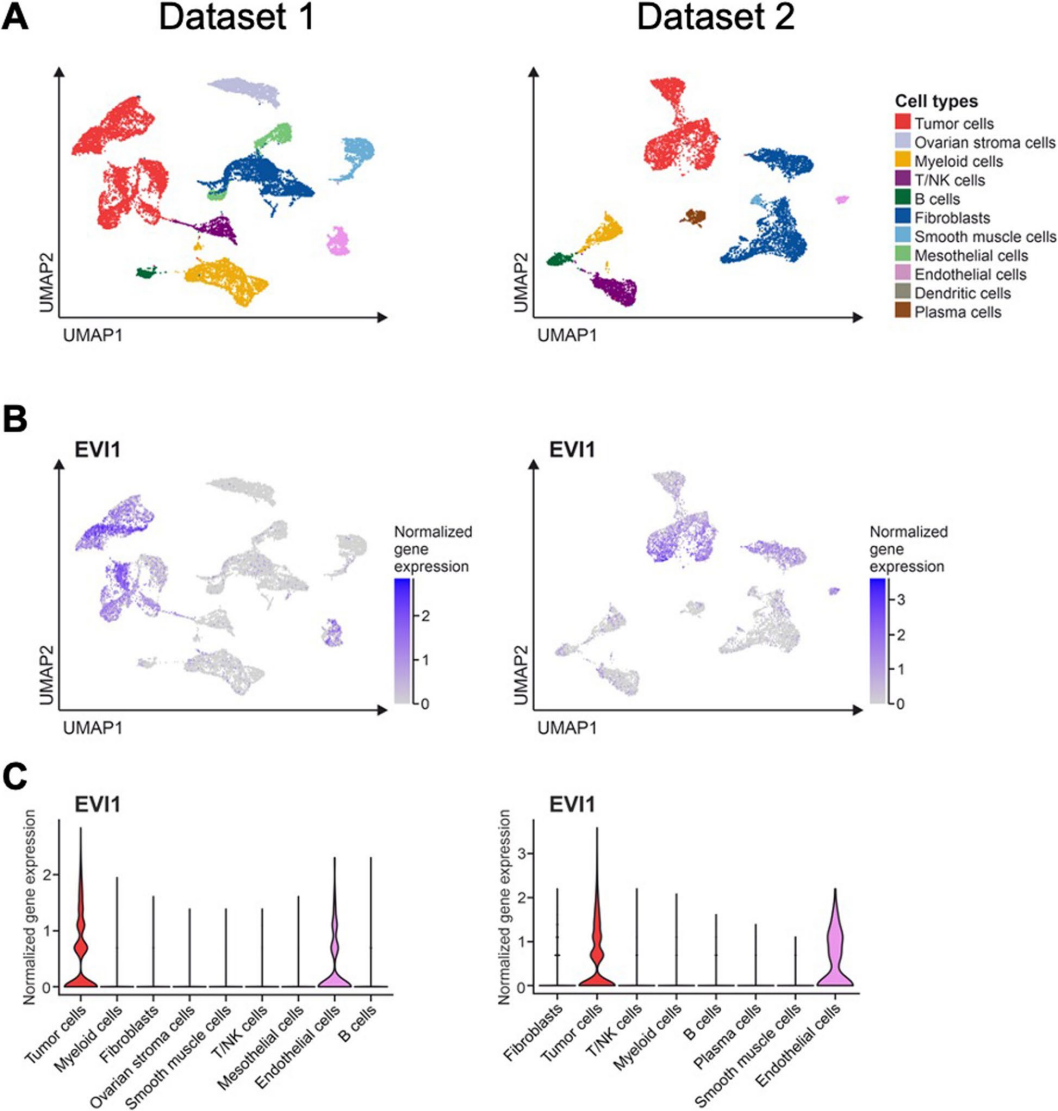
*EVI1* distribution in different cell types according to two publicly available single cell sequencing datasets from Olbrecht et al. (Dataset 1) and Olalekan et al. (Dataset 2) (**A**) Cell type distribution according to Uniform manifold approximation and projection (UMAP) (**B**) UMAP EVI1 distribution among different cell types (**C**) normalized EVI1 gene expression by cell types

By using single-cell sequencing data from Olbrecht et al. [31] and Olalekan et al. [32], we found that *EVI1* mRNA was highly expressed in tumor cells, thus confirming our immunohistochemical data. Figure 5A illustrates tumor cell clusters separately in both cohorts, while Fig. 5B shows *EVI1* mRNA distribution on clusters. *EVI1* was almost exclusively found in tumor cells with only a few tumor-associated fibroblasts and endothelial cells showing low mRNA levels.

## Discussion

We found that high EVI1 protein expression by immunohistochemistry is a robust prognostic biomarker for improved OS and PFS in HGSOC, especially in FIGO stages II-IV of HGSOC. Furthermore, we found an association with PARP1 protein expression by immunohistochemistry and expression of both markers identifying a subgroup with especially good prognosis.

To proof our findings, we confirmed *EVI1* expression on mRNA level in tumor cells with single-cell analyses of in silico data sets. With cBioPortal, we could confirm the association of *EVI1* and *PARP1* on mRNA level as well as on protein level. Regarding functional analysis, we did not find a common pathway involving both genes, but a common contribution to the MOTIF process of nuclear signal localization and to apoptosis.

While the prognostic impact of PARP1/2 is widely acknowledged, the role of EVI1 has not been described explicitly in a real-world dataset so far. *EVI1 *in vivo analyses, especially in ovarian carcinoma, are contradictory: some find it to be associated with worse survival, while others detect an association with better survival, although *EVI1* mRNA level was high in stage III/IV SOCs [35]. An increased copy number of 3q26.2 containing *EVI1* and *MDS1* genes with special emphasis on *EVI1* could be detected by different authors. On the one hand, Nanjundan et al. showed in a cohort of 62 patients, that gained *EVI1* DNA copy numbers are associated with better survival of patients with serous ovarian cancer (SOC) [36]. On the other hand, Österberg et al. described in a cohort of 40 stage III SOCs the negative prognostic effect of 3q26.2 gain being significantly associated with higher *EVI1* expression levels [35]. Although the combinatorial gene locus with *MECOM* has been found to be amplified in ovarian carcinoma, relevance of *EVI1* alone or in fusion with *MECOM* has still to be defined [5].

Using pancreatic cell lines and mouse models, Kim et al. recently showed that *EVI1* promotes tumor cell growth and motility in vitro and enhances tumor progression in vivo [37]. Using data from AML patients, Gröschel et al. showed the same poor influence of high *EVI* on disease remission and patients’ survival [25].

Apart from analyzing mRNA with the commonly known confounders of bulk tissue analysis and posttranslational gene modification, the cited studies have in common that diagnostic criteria for separation between high- and low-grade serous carcinomas were not specified. Low- and high-grade serous ovarian carcinomas were first recognized as different entities rather than low- and high-grade forms of the same neoplasm by the WHO classification in 2014 [3]. Furthermore, low-grade serous carcinoma is far less frequent than high-grade serous carcinoma impeding comparability of the above-mentioned studies among each other and with our results.

Analysis of PARP1/2 in ovarian carcinoma has been done thoroughly, and different studies have evaluated immunohistological PARP1/2 expression and its association with *BRCA* mutational status or survival and have found a positive correlation [38, 39]. High PARP1/2 expression discriminates patients who will respond appropriately or poorly to platinum-based chemotherapy [40, 41]. Few studies are in line with our findings and confirm this relation (high PARP1/2 expression = improved survival) [42]. Still, the role of PARP1 remains inconsistent until today, as e.g. Molnar et al. showed in a cohort of 104 HGSOC that high nuclear PARP1/2 expression is associated with worse patients’ outcome by using a polyclonal antibody [43]. While our *PARP1 *in silico analyses via KMp showed no prognostic influence on patients’ survival, the PARP1 protein expression analysis identified a patient subgroup with slightly improved survival rates and lower rates of PFS and OS events in the prediction models.

The in silico analyses confirmed our findings for EVI1: first, the expression in tumor cells could be proofed on the single cell level, and second, the positive influence on survival could be replicated on mRNA level in the Kaplan–Meier plotter.

Most interestingly, a high EVI1 protein expression was positively associated with high PARP1 protein expression in our cohort (*p* = 0.019). This association could also be detected in open-source datasets: using Spearman correlation analyses, the effect was found in independent cohorts on mRNA and protein level (MS by MS and RPPA by MS). However, pathway analysis (functional annotation chart) identified two common pathways contributing to the MOTIF process of nuclear localization signal and to apoptosis for *EVI1* and *PARP1*. We doubt these results due to the *p*-values of *p* = 1.0 adjusted to multiple testing. Therefore, we can only speculate about their synergistic biological function.

Interactions between *EVI1* and the *TGF-b* pathway as well as the *TGF-b* pathway with the cleavage of *PARP1* have been described [19]. Our idea to analyze EVI1 as a relevant protein in Fanconi anemia and thus in DNA repair mechanisms would hint at an interaction of EVI1 and PARP1 in this regard. Since many HGSOC lack homologous recombination, they depend on other DNA repair mechanisms, importantly also base excision repair via PARP1/2. Nevertheless, the explanation is difficult since we found a better survival for patients with high EVI1 and high PARP1 protein expression, which would imply protection of apoptosis of cancer cells and therefore rather expect worse survival. However, high EVI1 and PARP1 protein expression clearly mark a subgroup of good prognosis and since their mechanisms on disease progression are not yet fully understood, detailed modalities of interaction remain to be investigated.

Of note, a purely statistical association resulting in a third, not yet known regulation of both proteins should be kept in mind. Since 413 of 438 patients with available therapy data received a combined Taxol- and Platinum-based chemotherapy, no analysis stratified for therapy was conducted. Recently, therapy regimes for ovarian cancer have been extended by PARP inhibitors. Since this therapy concept is rather new, only few patients have received it yet and not in first line. But for further investigation, association of EVI1 with PARP1 and consecutive PARPi-therapy is a very interesting objective. This study was done retrospectively and no prospective validation of the data was performed. A further limitation is the small case number with FIGO I stage disease (*n* = 29), which could result in a statistical bias.

As a conclusion, our results show that EVI1 and PARP1 protein expression, used singly or combined, are promising robust and practical prognostic biomarkers regarding HGSOC patients’ survival. Based on our results, the role of EVI1 in HGSOC as well as its biological interaction with PARP1 and PARPi therapy should be further evaluated.

## Supplementary Information

**Additional file 1:** **Suppl. Fig. 1. **Systematic analysis for optimal cut-off for PARP1 expression on tumor cells as prognostic marker in HGSOC. (A) Frequency of PARP1 values (B) Hazard ratio (HR) and 95% confidence intervals (CI) of continuous PARP1 expression using overall survival in biostatistical tool *Cutoff Finder.*

**Additional file 2:** **Suppl. Fig. 2.** Survival analysis in PARP1 low and high HGSOC tumors, with and without FIGO stratification. (A) uni- and multivariate cox proportional hazards models with binary biomarker category (B) Kaplan-Meier plots and differences in median survival.

**Additional file 3:** **Suppl. Fig. 3.** Kaplan-Meier curves for PFS and OS using combined EVI1-PARP1 protein expression profiles.

**Additional file 4:** **Suppl. Fig. 4.** *In-silico *survival analysis of EVI1 and PARP1 mRNA expression profiles using Affymetrix gene-chip data. For analysis, patients with grade 2 + 3 HGSOC were split by the best cutoff method with a follow up threshold of 120 months.

**Additional file 5:** **Suppl. Fig. 5. **In-silico analysis of *EVI1* and *PARP1* using different level. (A) Copy number level (B) mRNA level (C and D) protein level (E) functional annotation chart.

**Additional file 6:** **Suppl. Tab. 1. **QuPath analysis parameters.

## Data Availability

All relevant data are within the paper and its Supporting Information files. The data underlying the results presented in the study are available from the study group, some restrictions apply due to confidentiality of patient data. Since these data are derived from a retrospective research trial with ongoing follow up, there are legal and ethical restrictions to share sensitive patient related data publicly. Data can be requested in the context of a translational research project by sending a request to the corresponding author.
