# Supplementary figures and images for: High EVI1 and PARP1 expression as favourable prognostic markers in high-grade serous ovarian carcinoma
Source: J Ovarian Res. 2023 Jul 31;16:150. doi: 10.1186/s13048-023-01239-6 (PMC10388497; doi:10.1186/s13048-023-01239-6)

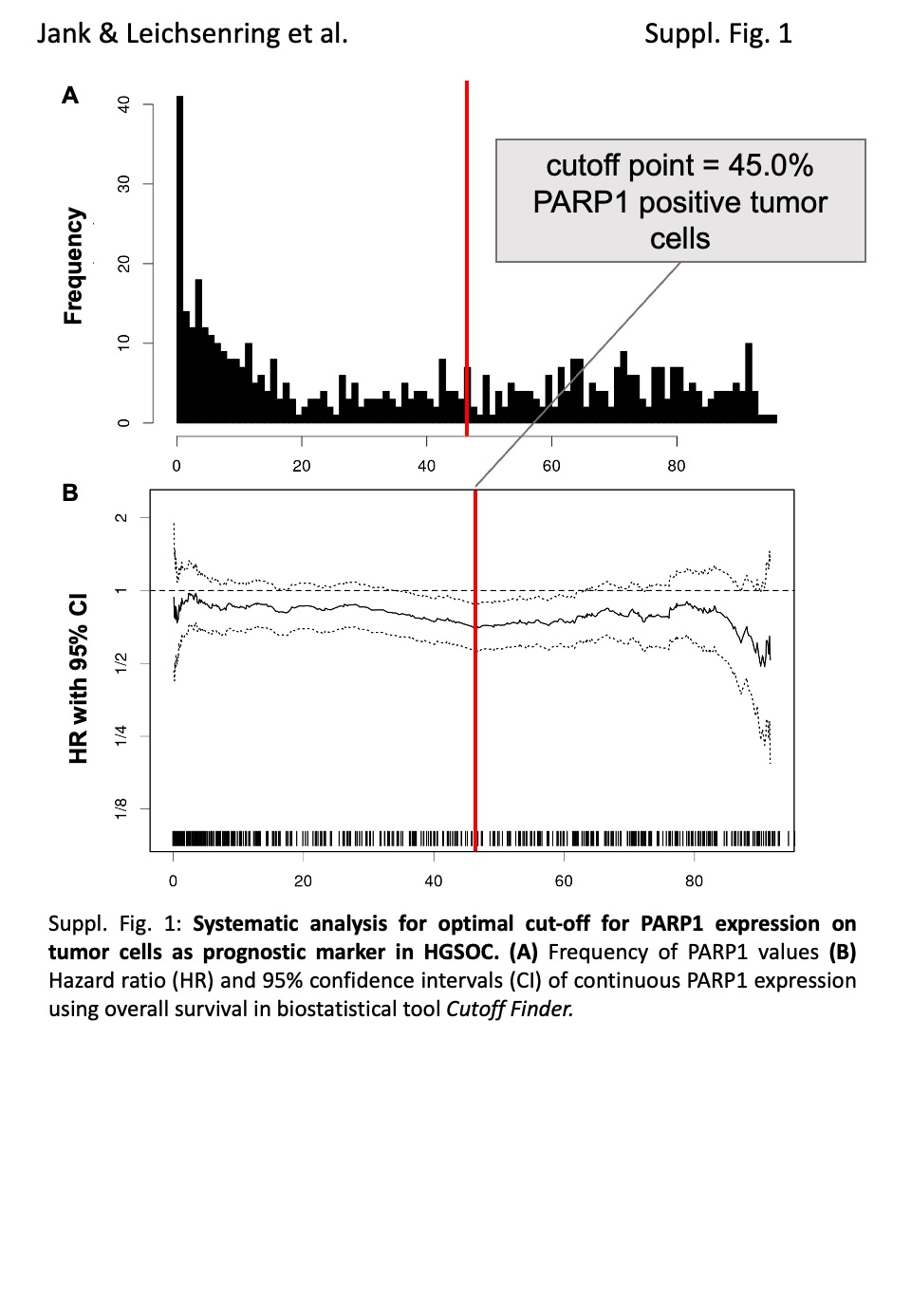

Supplement: Supplementary file 1 — Additional file 1: Suppl. Fig. 1. Systematic analysis for optimal cut-off for PARP1 expression on tumor cells as prognostic marker in HGSOC. (A) Frequency of PARP1 values (B) Hazard ratio (HR) and 95% confidence intervals (CI) of continuous PARP1 expression using overall survival in biostatistical tool Cutoff Finder. [file 13048_2023_1239_MOESM1_ESM.jpeg]

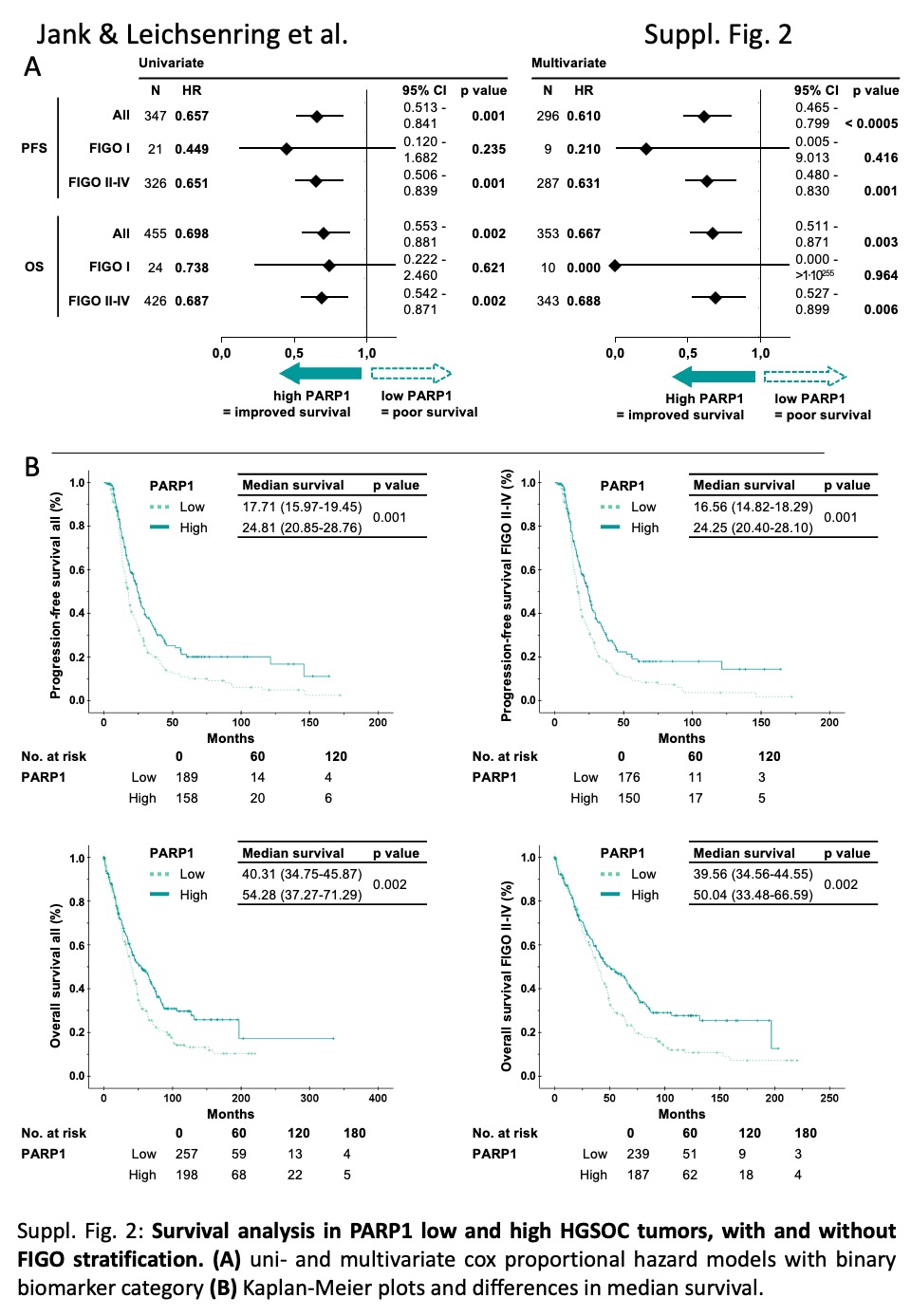

Supplement: Supplementary file 2 — Additional file 2: Suppl. Fig. 2. Survival analysis in PARP1 low and high HGSOC tumors, with and without FIGO stratification. (A) uni- and multivariate cox proportional hazards models with binary biomarker category (B) Kaplan-Meier plots and differences in median survival. [file 13048_2023_1239_MOESM2_ESM.jpeg]

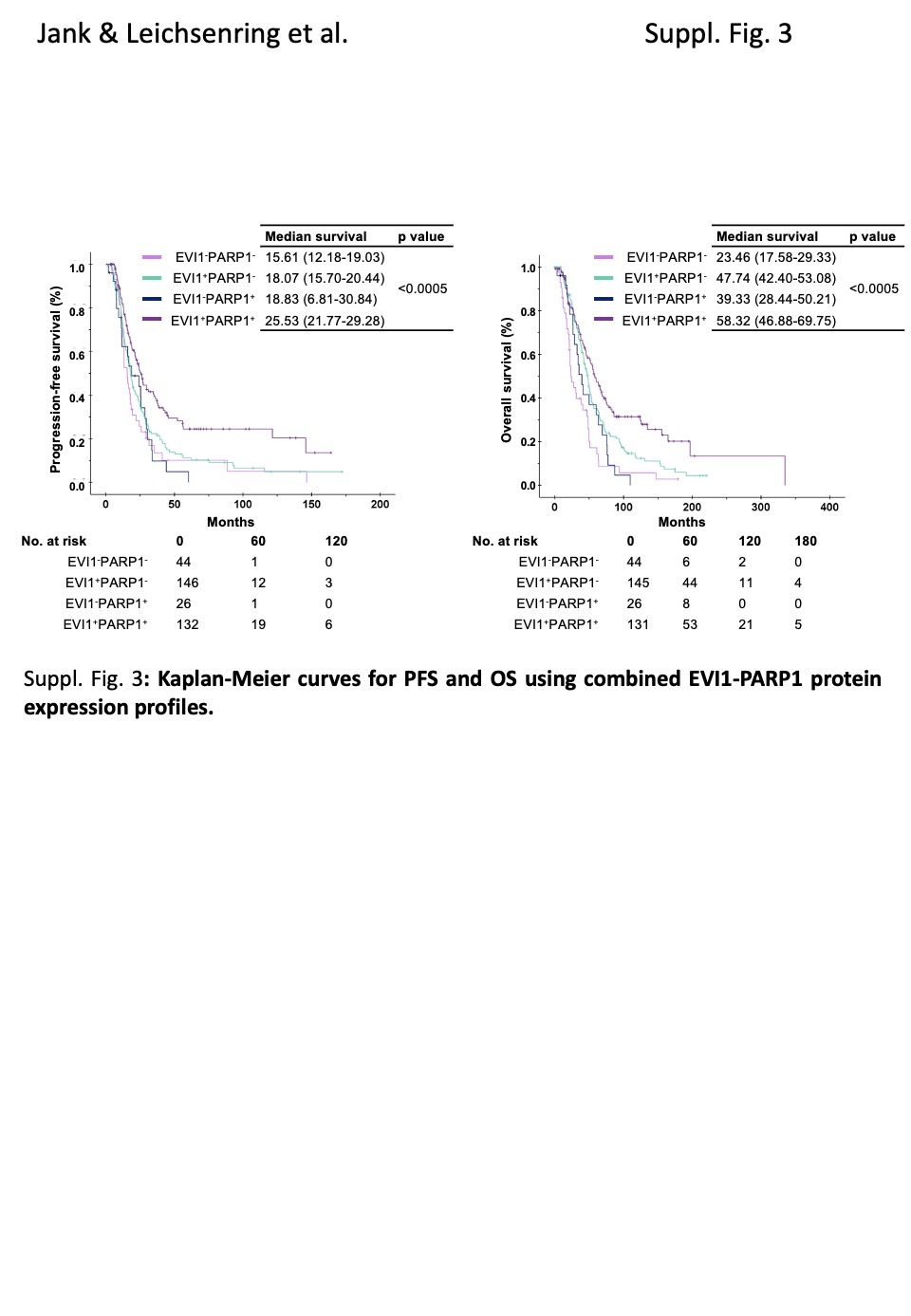

Supplement: Supplementary file 3 — Additional file 3: Suppl. Fig. 3. Kaplan-Meier curves for PFS and OS using combined EVI1-PARP1 protein expression profiles. [file 13048_2023_1239_MOESM3_ESM.jpeg]

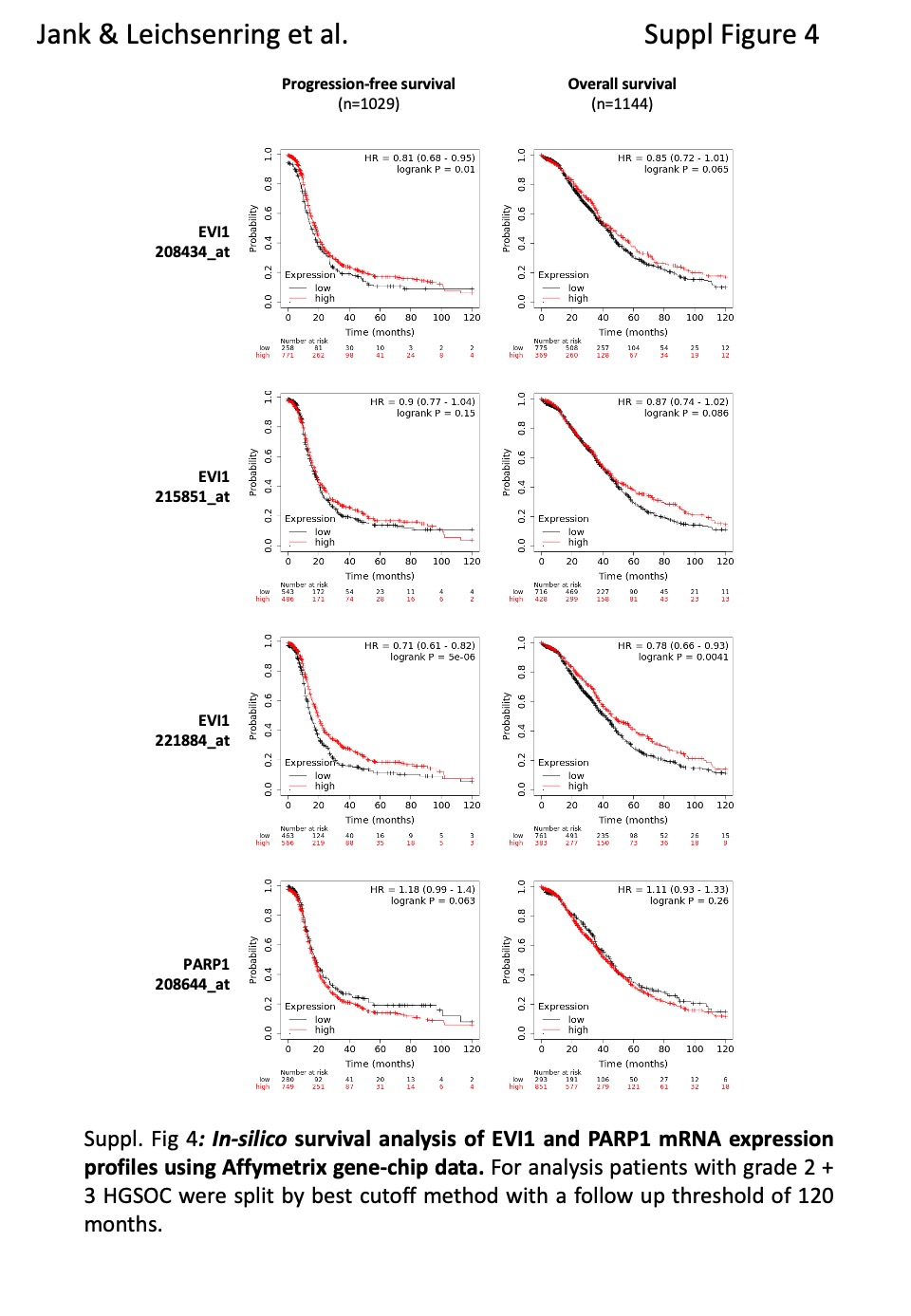

Supplement: Supplementary file 4 — Additional file 4: Suppl. Fig. 4. In-silico survival analysis of EVI1 and PARP1 mRNA expression profiles using Affymetrix gene-chip data. For analysis, patients with grade 2 + 3 HGSOC were split by the best cutoff method with a follow up threshold of 120 months. [file 13048_2023_1239_MOESM4_ESM.jpeg]

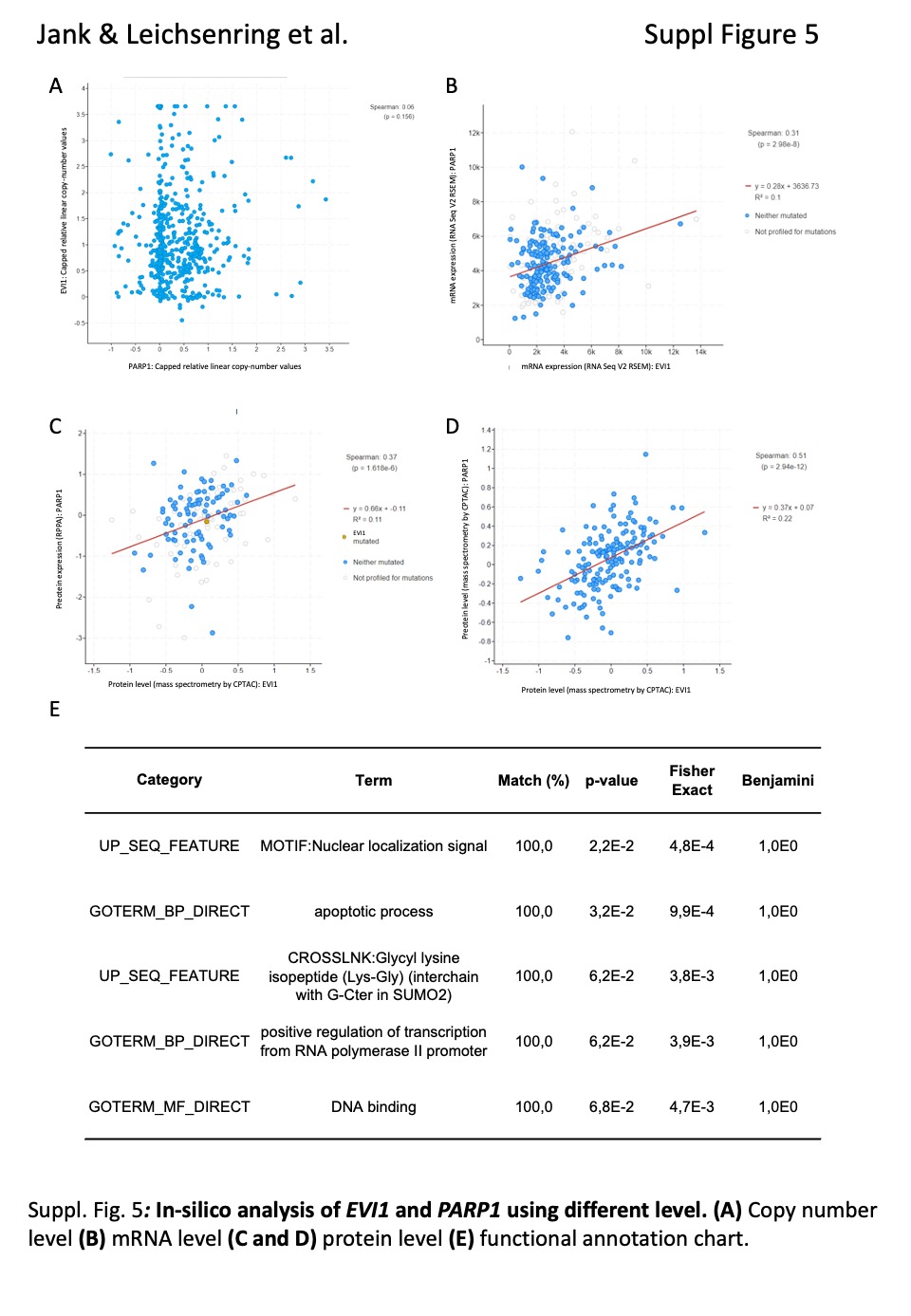

Supplement: Supplementary file 5 — Additional file 5: Suppl. Fig. 5. In-silico analysis of EVI1 and PARP1 using different level. (A) Copy number level (B) mRNA level (C and D) protein level (E) functional annotation chart. [file 13048_2023_1239_MOESM5_ESM.jpeg]

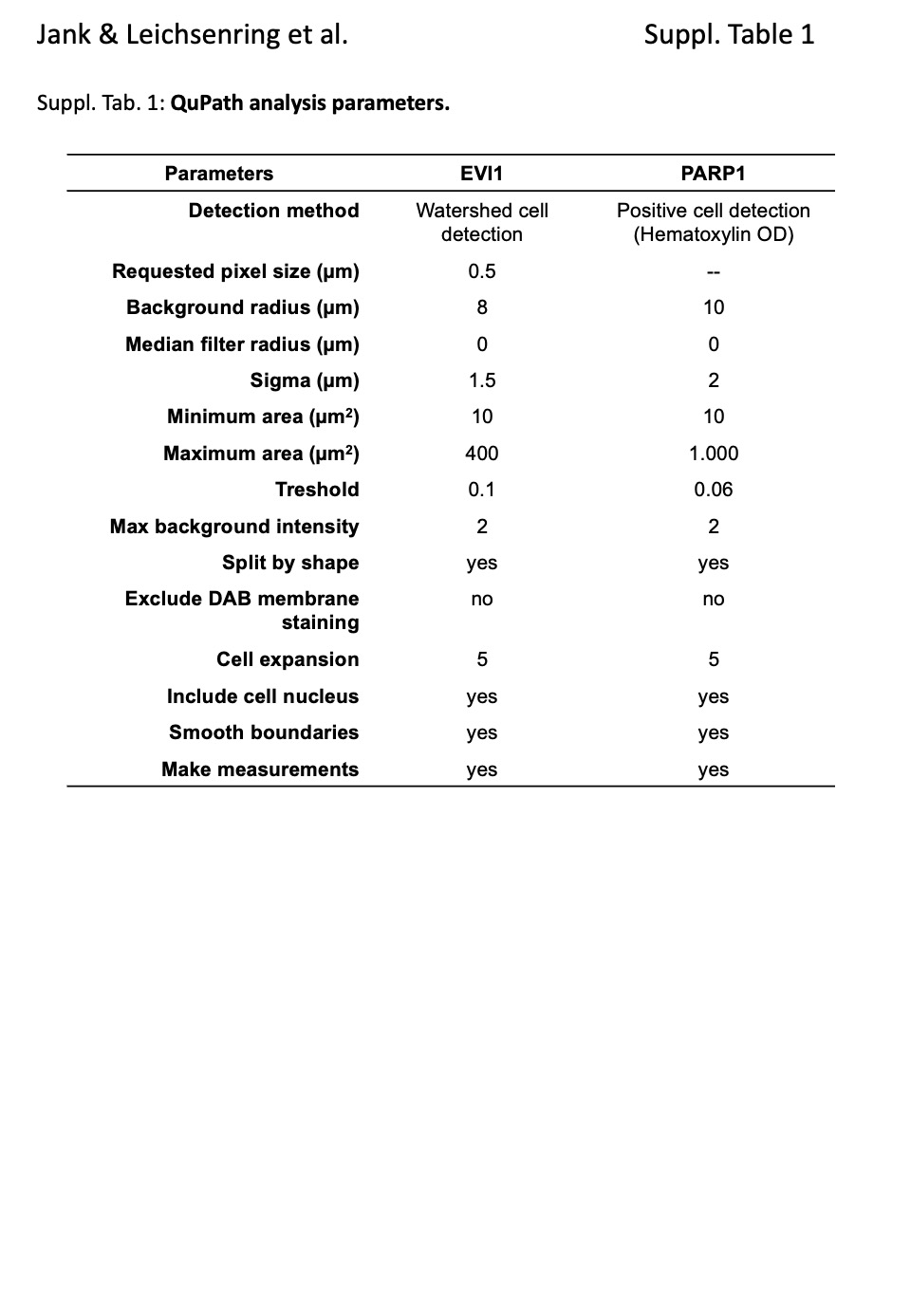

Supplement: Supplementary file 6 — Additional file 6: Suppl. Tab. 1. QuPath analysis parameters. [file 13048_2023_1239_MOESM6_ESM.jpeg]
